# Effects of Dietary and Probiotic Interventions in Patients with Metabolic Syndrome and Obstructive Sleep Apnea

**DOI:** 10.3390/clinpract15090159

**Published:** 2025-08-29

**Authors:** Amina Venter, Amin-Florin El-kharoubi, Mousa El-kharoubi, Evelin Claudia Ghitea, Marc Cristian Ghitea, Timea Claudia Ghitea, Ciprian Florian Venter

**Affiliations:** 1 Doctoral School of Biological and Biomedical Sciences, University of Oradea, 410087 Oradea, Romania; aminaadnan2005@yahoo.com; 2 Bihor Clinical County Emergency Hospital, 410169 Oradea, Romania; amin_kharubi@yahoo.com (A.-F.E.-k.); cypry_85@yahoo.com (C.F.V.); 3 The County Emergency Clinical Hospital of Târgu Mureș, 540136 Târgu Mureș, Romania; elkharoubimousa@gmail.com; 4 Faculty of Medicine and Pharmacy, University of Oradea, 410068 Oradea, Romania; ghitea.evelinclaudia@gmail.com (E.C.G.); ghitea.marc@gmail.com (M.C.G.); 5 Pharmacy Department, Faculty of Medicine and Pharmacy, University of Oradea, 410068 Oradea, Romania

**Keywords:** metabolic syndrome, obstructive sleep apnea, diet therapy, probiotics, GABA, glutamate

## Abstract

**Background:** Metabolic syndrome (MS) and obstructive sleep apnea (OSA) frequently coexist, exacerbating systemic inflammation, oxidative stress, and metabolic dysregulation. This study evaluates the effects of dietary and probiotic interventions, compared to a non-intervention control group, on metabolic, hemodynamic, and neurochemical parameters, with a specific focus on the neurotransmitters GABA and glutamate. **Methods:** In a prospective randomized study (2020–2023), 120 patients with coexisting MS and OSA were assigned to three groups: control (*n* = 36), diet therapy (*n* = 42), and diet therapy combined with probiotics (*n* = 42). Interventions lasted six months and included personalized dietary plans and probiotic supplementation. Outcome measures included BMI, visceral fat, HOMA index, lipid profile, oxygen saturation, and urinary GABA and glutamate levels. Unsupervised K-means clustering and principal component analysis (PCA) were applied to identify phenotypic response patterns based on delta values. **Results:** Diet therapy led to significant reductions in BMI (−15.7%, *p* = 0.001), visceral fat (−17.3%, *p* = 0.001), triglycerides (−14.6%, *p* = 0.003), uric acid (−9.5%, *p* = 0.011), and C-reactive protein (CRP) (−21.4%, *p* = 0.007). The combined intervention group exhibited further improvements in visceral fat (−22.8%, *p* = 0.001), glutamate (−18.2%, *p* = 0.002), and GABA levels (+19.5%, *p* = 0.001). Oxygen saturation improved across all groups, with the greatest increase in the probiotics group (+2.3%). Clustering analysis revealed three distinct response phenotypes—strong, moderate, and non-responders—highlighting inter-individual variability in treatment efficacy. **Conclusions:** Personalized dietary interventions, especially when paired with probiotics, effectively improve metabolic, inflammatory, and neurochemical profiles in patients with MS and OSA. Integrating clustering algorithms enables phenotype-specific stratification, offering a step toward precision lifestyle medicine. Future studies should explore long-term outcomes and refine microbiota-targeted approaches to optimize intervention efficacy.

## 1. Introduction

### 1.1. Pathophysiological Mechanisms Linking Metabolic Syndrome and Obstructive Sleep Apnea

Metabolic syndrome (MS) and obstructive sleep apnea (OSA) share complex pathophysiological mechanisms that exacerbate each other’s progression [1]. OSA is characterized by recurrent episodes of upper airway obstruction during sleep, leading to intermittent hypoxia and sleep fragmentation [2]. These events trigger a cascade of metabolic disturbances, including sympathetic nervous system overactivation, systemic inflammation, and oxidative stress [3]. Chronic intermittent hypoxia (CIH) contributes to insulin resistance, dyslipidemia, and hypertension, all core features of MS [4]. Simultaneously, MS predisposes individuals to OSA through increased fat deposition, particularly in the upper airway and visceral adipose tissue, which exacerbates airway collapsibility and respiratory disturbances [5].

The key pathophysiological mechanisms involved in MS and OSA include the following. Chronic Intermittent Hypoxia (CIH): Repeated oxygen desaturation and reoxygenation cycles lead to systemic oxidative stress and inflammation [6]. This process upregulates pro-inflammatory cytokines, such as tumor necrosis factor-alpha (TNF-α) and interleukin-6 (IL-6), contributing to endothelial dysfunction and insulin resistance [7].

Sympathetic Nervous System Overactivity: OSA-induced hypoxia triggers increased sympathetic tone, elevating blood pressure, heart rate, and systemic vascular resistance, which further deteriorates metabolic homeostasis [8].

Neurotransmitter Imbalance: Gamma-aminobutyric acid (GABA) and glutamate, crucial regulators of metabolic and neurophysiological function, are disrupted in OSA and MS [9]. A decline in GABA levels reduces its inhibitory effects on inflammation and metabolic stress, whereas elevated glutamate levels promote excitotoxicity and metabolic dysregulation [10].

Gut Microbiota Dysbiosis: Alterations in the gut microbiota composition due to MS and OSA can influence metabolism and systemic inflammation. Probiotics have shown potential in modulating the gut microbiota to restore metabolic balance and improve neurotransmitter regulation [11].

Adipose Tissue Dysfunction and Systemic Inflammation: Increased visceral adiposity in MS exacerbates systemic inflammation, releasing adipokines such as leptin and resistin while suppressing adiponectin, further increasing metabolic disturbances and worsening OSA severity [12].

Hormonal Dysregulation: Dysregulation of insulin, cortisol, and ghrelin plays a critical role in both MS and OSA, leading to persistent metabolic impairments and increased cardiovascular risk [13]. Understanding these mechanisms is crucial for developing integrated therapeutic strategies that address both conditions simultaneously.

Sleep apnea syndrome (SAS) is among the most prevalent sleep-related breathing disorders, affecting 9% to 38% of the general population, with a significantly higher prevalence observed in individuals with MS and obesity [14,15]. SAS is characterized by recurrent episodes of hypopnea and OSA, which contribute to sleep fragmentation, intermittent hypoxemia, and elevated catecholamine release [16]. These pathophysiological processes negatively impact cardiovascular, metabolic, and cognitive function [17,18,19].

In patients with MS, SAS exacerbates the chronic inflammatory state and oxidative stress, which are pivotal in the development of insulin resistance and atherosclerosis [20]. This interplay between SAS and MS underscores the necessity of innovative therapeutic strategies targeting both metabolic dysfunction and neurohormonal imbalances [21].

The central nervous system is instrumental in the regulation of metabolism, with neurotransmitters such as GABA and glutamate playing critical roles. GABA, the principal inhibitory neurotransmitter, exerts anti-inflammatory and antioxidative effects, while also promoting sleep quality [22,23]. Conversely, glutamate, as an excitatory neurotransmitter, is associated with excitotoxicity under chronic stress conditions, disrupting metabolic homeostasis. An imbalance between GABA and glutamate is now seen as a key factor in the development of SAS and MS, opening up new possibilities for treatment through diet and medication [24]. Furthermore, the imbalance between the neurotransmitters GABA and glutamate is increasingly recognized as a potential factor in MS and OSA pathogenesis. Thus, addressing these neurochemical imbalances through dietary and probiotic interventions could provide a novel therapeutic strategy for the management of these conditions [25].

Dietary interventions, particularly when combined with probiotics, have shown promising results in reducing systemic inflammation and enhancing metabolic function [26]. Probiotics modulate the gut microbiota, which in turn regulates the production of metabolites and neurotransmitters such as GABA and glutamate, crucial for maintaining systemic homeostasis [27].

### 1.2. Rationale and Significance of the Study

Previous research has examined dietary and probiotic interventions in metabolic disorders, but few studies have comprehensively assessed their combined effects on both metabolic and neurochemical parameters in patients with MS and OSA [28]. While dietary modifications have been associated with metabolic improvements, results vary due to differences in diet composition and adherence [29]. Similarly, probiotics have demonstrated potential in modulating the gut microbiota, reducing inflammation, and improving metabolic markers, but their impact on neurotransmitter levels remains inconclusive [30].

**Figure 1:**
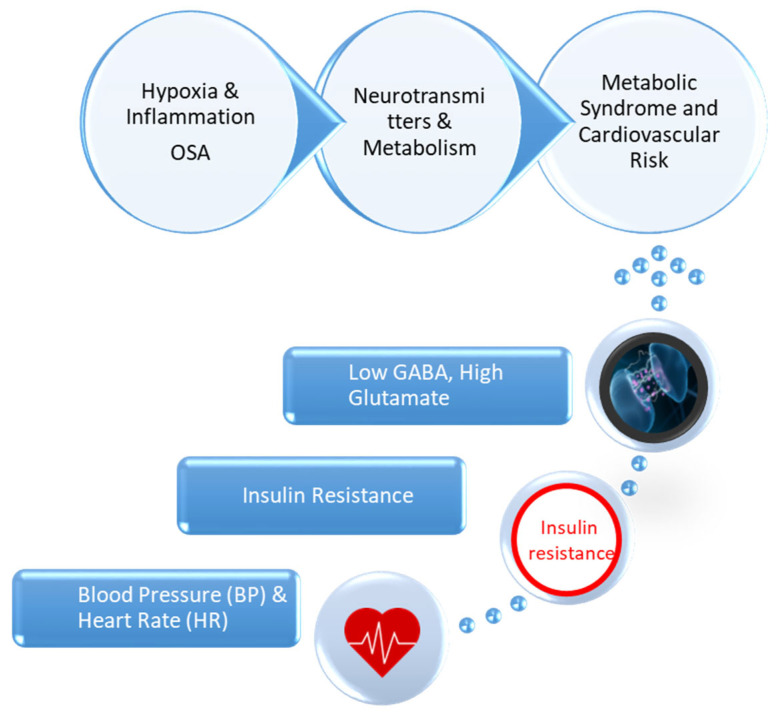
Interplay Between OSA, Neurotransmitter Imbalance, and Metabolic Dysfunction.

This study aims to address these gaps by evaluating how dietary and probiotic interventions influence metabolic, hemodynamic, and biochemical parameters, with a particular focus on GABA and glutamate. The specific objectives include assessing changes in body mass index (BMI), fat mass, visceral fat, Homeostasis Model Assessment (HOMA) index, cholesterol, triglycerides, and oxygen saturation before and after the interventions. Additionally, the study investigates the roles of GABA and glutamate as biomarkers of metabolic and neurophysiological regulation. By exploring the interactions among SAS, metabolic syndrome, and neurotransmitters, this research seeks to develop personalized treatment strategies. Identifying the relationships between dietary interventions, probiotics, and metabolic parameters may contribute to integrated approaches for the management of MS and OSA. Figure 1 presents the interplay between OSA, neurotransmitter imbalance, and metabolic dysfunction.

## 2. Materials and Methods

### 2.1. Study Design

A prospective, randomized study was conducted between 2020 and 2023 at a private nutrition clinic in Romania, adhering to the ethical principles outlined in the Declaration of Helsinki of the World Medical Association. The primary objective was to evaluate the effects of dietary and probiotic interventions on metabolic, hemodynamic, and biochemical parameters in patients diagnosed with MS and OSA, with a particular focus on the neurotransmitters GABA and glutamate. The study aimed to identify effective interventions to improve systemic health and metabolic function.

The sample size was calculated using GPower (version 3.1.9.7) for ANOVA with three groups, effect size f = 0.30 (medium), α = 0.05, and power = 0.80, requiring at least 111 participants. We included 120 to account for potential dropout.

### 2.2. Inclusion and Exclusion Criteria

Participants were selected based on strict eligibility criteria to ensure homogeneity within the study population. Eligible individuals were aged 18 to 65 years, diagnosed with MS and OSA, and had a body mass index (BMI) of 25 kg/m^2^ or higher. Written informed consent was obtained from all participants, who also agreed to comply with the study protocols. MS was defined using the IDF 2005 criteria. OSA was confirmed by polysomnography with AHI ≥ 15 (moderate to severe).

Metabolic syndrome was defined according to the International Diabetes Federation (IDF) criteria, requiring central obesity plus at least two of the following: elevated triglycerides, reduced HDL cholesterol, elevated blood pressure, or increased fasting glucose. Obstructive sleep apnea was diagnosed via polysomnography and classified based on the Apnea–Hypopnea Index (AHI), with all included patients having AHI ≥ 15, consistent with moderate to severe OSA.

**Figure 2:**
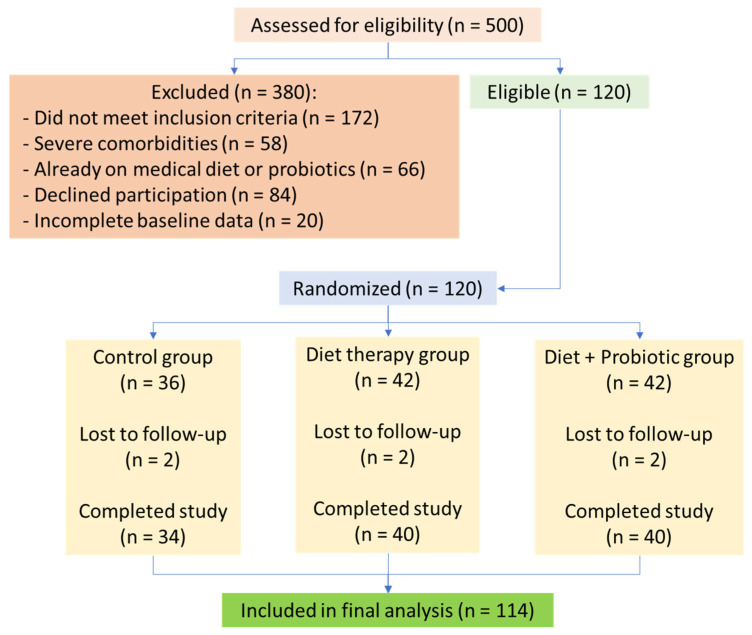
Flowchart of participant selection, allocation, follow-up, and analysis. From 500 screened patients with suspected metabolic syndrome and obstructive sleep apnea (OSA), 120 were enrolled and randomized into three groups. Attrition due to withdrawal or loss to follow-up was minimal (*n* = 6).

Patients with severe chronic diseases, such as renal or hepatic insufficiency, active neoplasms, or chronic gastrointestinal disorders, were excluded to minimize confounding variables that could obscure intervention effects. Although MS is associated with an increased risk of these severe conditions, the study aimed to evaluate dietary and probiotic interventions in a population with modifiable metabolic dysfunction, rather than those with advanced disease states requiring specialized medical management. Additionally, individuals on medications affecting neurotransmitter levels, pregnant or lactating women, and those unwilling to participate were excluded (Figure 2).

### 2.3. Participant Selection and Intervention

Participants were randomized via computer-generated block randomization (block size = 6) by an independent researcher. Allocation information was concealed in sealed opaque envelopes. From an initial pool of 500 patients assessed, 120 participants met the eligibility criteria and were randomly assigned to one of three groups.

Control Group (*n* = 36): No specific dietary or probiotic interventions; participants maintained their usual lifestyles.

Diet Therapy Group (*n* = 42): Received a personalized dietary plan designed to reduce metabolic risk. The diet emphasized increased intake of proteins, unsaturated fats, and fiber while limiting saturated fats, refined carbohydrates, and salt.

Diet Therapy and Probiotics Group (*n* = 42): Followed the same dietary plan as the diet therapy group but also received a standardized probiotic supplement containing bifidobacteria and lactobacilli (free from gluten and lactose). The probiotics were selected based on evidence supporting their roles in modulating the gut microbiota, reducing inflammation, and improving metabolic regulation.

Six participants discontinued the intervention (2 per group), resulting in a final sample of 114. Dropouts were due to personal reasons and not adverse events. Adherence was >85% based on logs and capsule counts.

The intervention lasted six months. Dietary adherence was monitored through food diaries and monthly consultations with a clinical nutritionist. Probiotic adherence was ensured through structured patient follow-ups. The necessity of combining diet with probiotics was based on growing evidence suggesting synergistic effects in improving metabolic and inflammatory profiles, particularly in patients with MS and OSA.

The probiotic supplement included standardized strains of Lactobacillus acidophilus and Bifidobacterium lactis, delivering a total of 10^9^ CFU per capsule. Participants were instructed to take one capsule daily with meals for six months. Adherence was monitored via monthly capsule counts and patient-reported logs.

### 2.4. Biochemical and Anthropometric Measurements

To ensure accuracy and standardization, all biochemical and anthropometric measurements were performed by trained medical professionals under controlled laboratory conditions.

For anthropometric assessments, body mass index (BMI), fat mass, and visceral fat were measured using bioelectrical impedance analysis (BIA), a validated and widely used method for the assessment of body composition.

The Homeostatic Model Assessment for Insulin Resistance (HOMA) index was calculated using the standard formula: HOMA-IR = (fasting insulin (µU/mL) × fasting glucose (mg/dL))/405.

Cholesterol and triglycerides were measured using automated enzymatic colorimetric assays, conducted in certified laboratories following international standards.

Hemodynamic markers (blood pressure and oxygen saturation) were assessed using a calibrated automated sphygmomanometer for blood pressure and a pulse oximeter for oxygen saturation, with measurements taken under standardized conditions.

Biochemical markers (GABA and glutamate levels) were quantified using high-performance liquid chromatography (HPLC) with fluorescence detection, a sensitive and precise method for neurotransmitter analysis.

Each test was conducted by specialized laboratory personnel trained in metabolic and biochemical analyses, ensuring consistency and reliability in sample processing.

### 2.5. Statistical Analysis

Statistical analyses were conducted using the SPSS software (version 30.0, IBM Corp., Armonk, NY, USA), with a significance level set at *p* < 0.05 [31].

Comparative Analyses: Paired *t*-tests were used to evaluate within-group differences before and after the intervention. One-way ANOVA with post hoc Bonferroni correction was applied to determine statistically significant differences among groups. Repeated-measures ANOVA was conducted to assess changes over time while accounting for individual variability. In addition to univariate and ANOVA analyses, we performed multiple linear regression to identify independent predictors of the HOMA index and C-reactive protein (CRP), adjusting for age, sex, baseline BMI, and oxygen saturation. Due to limited CPAP data, this variable could not be included in all models.

For normality, the Shapiro–Wilk test was used. For non-normal distributions, non-parametric tests (Mann–Whitney, Kruskal–Wallis) were used as appropriate.

Correlational Analyses: Spearman’s correlation coefficients were calculated to explore associations between neurotransmitter levels (GABA and glutamate) and metabolic parameters (BMI, visceral fat, HOMA index, cholesterol, triglycerides, and CRP). Multiple regression models were used to identify independent predictors of metabolic improvements in response to dietary and probiotic interventions.

The selection of statistical methods was based on recommendations from the established biostatistics literature to ensure methodological rigor. The analyses aimed to provide robust insights into the impacts of dietary and probiotic interventions on metabolic and biochemical markers.

### 2.6. Ethics Committee Approval

The study received ethical approval from the ethics committee of the private clinic, under protocol number CEFMF/12/01.04.2019 and CEFMF/1/31.10.2024. Written informed consent was obtained from all participants before study enrollment. The research was conducted in full compliance with ethical standards, ensuring participant safety and data confidentiality.

### 2.7. Clustering Algorithm

**Figure 3:**
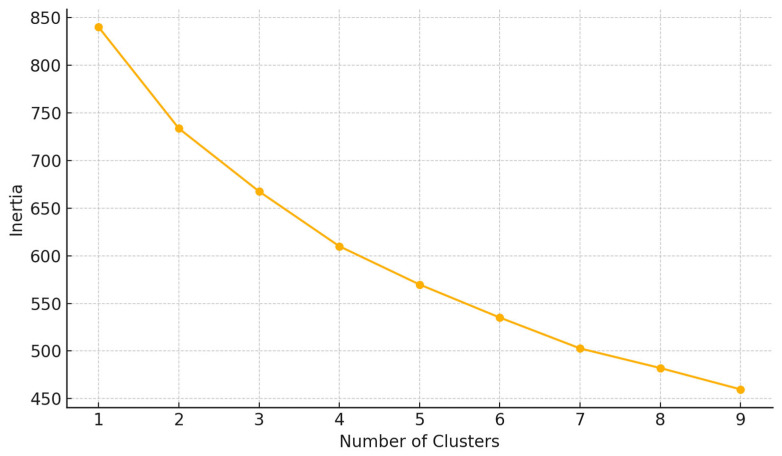
Elbow method for optimal clusters.

We used a K-means clustering to identify subgroups (“responders”, “partial responders”, “non-responders”) based on improvements in key markers (Figure 3).

Variables used for clustering: BMI, visceral fat, HOMA index, GABA, glutamate, CRP, oxygen saturation.

Cluster 0: Strong responders (marked improvements in BMI, GABA ↑, CRP ↓);

Cluster 1: Partial responders (some metabolic changes, minor neurotransmitter shifts);

Cluster 2: Non-responders (minimal improvements).

## 3. Results

### 3.1. Demographic and Group Characteristics

A total of 120 participants were included in the study and divided into three groups: the control group, the diet therapy group, and the diet therapy combined with probiotics group. The distribution based on the area of residence revealed a predominance of patients from rural areas, particularly in the intervention groups, while urban residents were more frequently represented in the control group. This uneven distribution may reflect differing levels of access to or interest in lifestyle interventions between rural and urban populations.

Regarding the gender distribution, men were more prevalent than women across all groups, suggesting a possible gender-related factor in the recruitment or prevalence of the conditions studied. The average age of participants was notably lower in the diet therapy and probiotics group compared to the other groups. This trend may indicate higher willingness or a predisposition among younger individuals to engage in lifestyle-based interventions, possibly driven by health awareness or motivation to prevent disease progression at an earlier stage. The demographic and group characteristics of study participants is presented in Table 1, and the extended table is presented in Table S1 (Supplementary Materials).

### 3.2. Metabolic Parameters

Table 2 presents a comparison of the metabolic, biochemical, and hemodynamic parameters among the three study groups—control, diet therapy, and diet therapy with probiotics—at baseline (Initial) and post-intervention (Final). The mean values and standard deviations (SDs) for each parameter are displayed, alongside *p*-values indicating statistical significance.

#### 3.2.1. Body Composition and Metabolic Markers

At baseline, the body mass index (BMI) was comparable across groups, with the control group averaging 29.29 (SD = 5.60), the diet therapy group at 31.03 (SD = 6.28), and the diet therapy with probiotics group at 31.42 (SD = 9.89). Following the intervention, the BMI remained largely unchanged in the control group (29.47, SD = 5.79) but significantly improved in both intervention groups, with the most notable reduction in the diet therapy with probiotics group (24.77, SD = 3.19, *p* = 0.001).

Similarly, fat mass and visceral fat followed a comparable trend, with the control group showing no meaningful reduction, while the diet therapy and probiotics group exhibited the greatest improvement (fat mass: 20.75, SD = 7.86; visceral fat: 3.90, SD = 2.77, *p* = 0.001).

#### 3.2.2. Glycemic Control and Insulin Resistance

Baseline fasting blood glucose (FBG) values were highest in the diet therapy with probiotics group (103.68, SD = 18.98), followed by the control group (101.5, SD = 31.12) and the diet therapy group (94.1, SD = 23.81). Post-intervention, FBG improved significantly, particularly in the probiotics group (81.80, SD = 14.98, *p* = 0.009), indicating better glycemic control.

Insulin levels increased in the control group post-intervention (17.1, SD = 4.12), whereas both intervention groups experienced reductions, with the best outcome in the probiotics group (9.16, SD = 5.14, *p* = 0.007). Similarly, the HOMA index, a key indicator of insulin resistance, improved most in the probiotics group (2.29, SD = 1.50, *p* = 0.001), suggesting a positive effect of the combined dietary and probiotic intervention.

#### 3.2.3. Lipid Profile and Inflammatory Markers

Baseline HDL cholesterol (HDL-c) levels were lowest in the control group (32, SD = 5.66) and highest in the diet therapy group (51, SD = 5.16). Post-intervention, HDL-c increased significantly in both intervention groups, with the probiotics group achieving the highest levels (60, SD = 4.84, *p* = 0.001), indicating improved cardiovascular health.

Conversely, LDL cholesterol (LDL-c) and triglycerides (TG) followed the opposite pattern, with the control group showing an increase (LDL-c: 141.45, SD = 32.7; TG: 118.45, SD = 37.48), while both intervention groups exhibited reductions, with LDL-c decreasing most in the probiotics group (110, SD = 40.6, *p* = 0.001) and TG reaching its lowest value in the probiotics group (95, SD = 37.48, *p* = 0.001).

C-reactive protein (CRP), a marker of systemic inflammation, was lowest in the control group at baseline (0.46, SD = 0.25) but increased post-intervention (0.53, SD = 0.29), whereas both intervention groups experienced a reduction, with the probiotics group demonstrating the most significant improvement (0.30, SD = 0.27, *p* = 0.012).

#### 3.2.4. Blood Pressure and Cardiovascular Parameters

The baseline systolic and diastolic blood pressure was highest in the diet therapy group (141/100 mmHg), followed by the control group (132/97 mmHg). After six months, the control group showed worsening blood pressure parameters (151/107 mmHg), while both intervention groups demonstrated improvements, with the probiotics group achieving the most notable reductions (121/85 mmHg, *p* = 0.032 and 0.004, respectively).

The study findings demonstrate that diet therapy combined with probiotics led to the most significant improvements across all metabolic, glycemic, lipid, and inflammatory parameters. The control group exhibited worsening in most parameters, while the diet therapy group improved moderately, and the diet therapy with probiotics group showed the most substantial benefits. These results emphasize the potential role of dietary and probiotic interventions in managing MS and associated complications.

### 3.3. Hemodynamic Parameters

Table 3 summarizes the effects of different interventions on oxygen saturation and hypertension (HTN) across the three study groups: control, diet therapy, and diet therapy with probiotics. Oxygen saturation improved across all groups, with the highest increase in the probiotics group (98.80 ± 1.13) compared to the control (96.56 ± 1.92) and diet therapy groups (97.11 ± 2.04). At baseline, HTN was most prevalent in the control group (29 patients, 15.1%) and least in the probiotics group (20 patients, 10.4%), while, post-intervention, HTN significantly decreased in the intervention groups, especially in the probiotics group, where the proportion of patients with hypertension dropped from 10.4% to 2.1%. The total number of patients with normal blood pressure increased from 52.1% to 83.3%, demonstrating the beneficial effects of diet therapy, particularly when combined with probiotics, on cardiovascular health.

**Figure 4:**
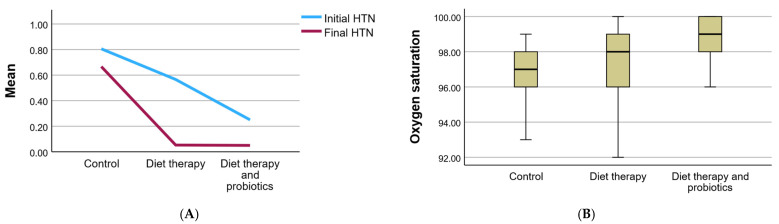
Hemodynamic parameters (HTN) before and after intervention across study groups (**A**) and oxygen saturation at baseline (**B**).

Significant improvements in hemodynamic parameters were observed in the intervention groups (Figure 4). Blood pressure showed marked reductions in the prevalence of hypertension (HTN). In the diet therapy group, the number of patients with HTN decreased from 44 to 4, while, in the diet therapy and probiotics group, the prevalence dropped from 20 to 4. These results indicate the effectiveness of dietary interventions, particularly when combined with probiotics, in managing blood pressure and reducing cardiovascular risk.

Oxygen saturation, a critical parameter reflecting respiratory efficiency, improved significantly across all groups. The most substantial increase was observed in the diet therapy and probiotics group, where the mean oxygen saturation rose from 96.56 ± 1.92 to 98.80 ± 1.13. This finding suggests an enhancement in respiratory function, likely mediated by the combined benefits of dietary changes and the modulation of the gut microbiota through probiotics. These hemodynamic improvements underscore the potential of integrated lifestyle interventions in addressing both cardiovascular and respiratory complications associated with MS and OSA.

### 3.4. Biochemical Parameters

**Figure 5:**
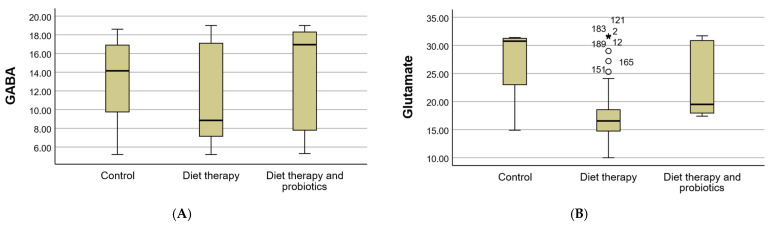
Distribution of GABA and glutamate levels across study groups. This figure presents box plots illustrating the distribution of the GABA (**A**) and glutamate (**B**) levels in the control, diet therapy, and diet therapy combined with probiotics groups. The plots highlight variations in neurotransmitter levels across groups, with diet therapy and probiotics showing notable changes. Outliers in the glutamate distribution are also marked for reference. Open circles indicate mild outliers (>1.5 × IQR), and asterisks denote extreme outliers (>3 × IQR); numbers correspond to individual observation IDs.

GABA levels exhibited a slight increase in both the control and probiotic groups, with the most notable improvement observed in the diet therapy and probiotics group, where the levels rose from 11.07 ± 5.03 to 13.84 ± 5.31. This finding suggests a potential positive impact of probiotics on metabolic regulation, possibly through the modulation of the neurotransmitter balance (Figure 5A).

Conversely, glutamate levels showed a significant decrease in the intervention groups. In the diet therapy and probiotics group, glutamate levels were moderately reduced, from 23.77 ± 6.14 to values approaching the normal range (Figure 5B). This reduction highlights the potential of combined interventions to mitigate excitotoxicity and restore metabolic homeostasis. Together, these biochemical changes reflect the beneficial effects of dietary and probiotic interventions on neurotransmitter regulation in the context of MS and OSA.

The distribution of participants by GABA and glutamate categoryies across study groups is presented in Table 4.

### 3.5. Cholesterol, Triglycerides, and Inflammatory Markers

Cholesterol and triglyceride levels improved significantly in both intervention groups, with the most pronounced reductions observed in the diet therapy and probiotics group, suggesting a potential decrease in cardiovascular risk. CRP, an important marker of systemic inflammation, decreased significantly in the intervention groups, indicating reduced inflammation. These findings reflect changes in biochemical parameters, including cholesterol, triglycerides, uric acid, and CRP, across the three study groups.

The control group showed minimal changes in all parameters, highlighting the limited influence of natural factors or non-specific interventions on metabolic and inflammatory markers. By contrast, diet therapy alone demonstrated the greatest impact in reducing cholesterol (−0.36 ± 0.56 − 0.36 ± 0.56 − 0.36 ± 0.56) and triglycerides (−0.33 ± 0.50 − 0.33 ± 0.50 − 0.33 ± 0.50), underscoring its effectiveness in improving lipid profiles. Diet therapy also significantly reduced uric acid levels (−0.28 ± 0.45 − 0.28 ± 0.45 − 0.28 ± 0.45) and systemic inflammation, as measured by CRP (−0.18 ± 0.39 − 0.18 ± 0.39 − 0.18 ± 0.39).

In the diet therapy and probiotics group, the effects were more variable. A slight reduction in triglycerides (−0.07 ± 0.27 − 0.07 ± 0.27 − 0.07 ± 0.27) and uric acid (−0.05 ± 0.22 − 0.05 ± 0.22 − 0.05 ± 0.22) was observed, but these changes were less pronounced compared to the diet therapy group. Additionally, the cholesterol levels in this group increased slightly (+0.20 ± 0.40 + 0.20 ± 0.40 + 0.20 ± 0.40), suggesting that probiotics may interact differently with lipid metabolism. Inflammation, as measured by CRP, was reduced to a lesser degree in the diet therapy and probiotics group (−0.11 ± 0.32 − 0.11 ± 0.32 − 0.11 ± 0.32) than in the diet therapy group, indicating a relatively limited effect of probiotics in reducing systemic inflammation.

**Figure 6:**
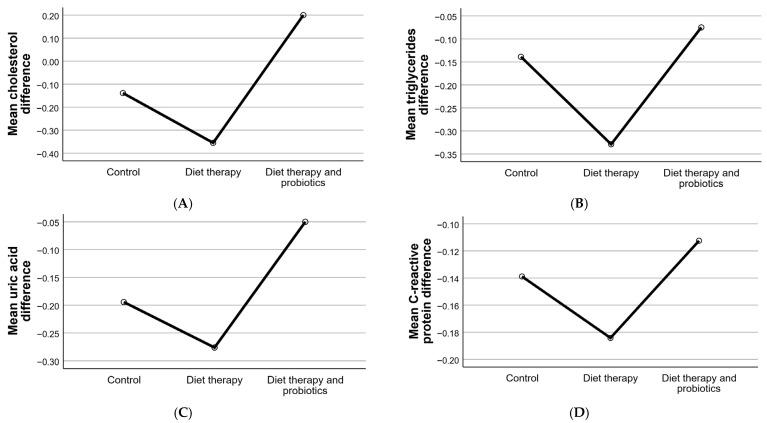
Changes in biochemical parameters across study groups. These graphs illustrate the mean differences (final − initial) in key biochemical parameters, including cholesterol (**A**), triglycerides (**B**), uric acid (**C**), and C-reactive protein (CRP) (**D**), across the control, diet therapy, and diet therapy with probiotics groups. The trends highlight the distinct effects of the interventions on metabolic and inflammatory markers.

These findings suggest that diet therapy alone is the most effective intervention in reducing metabolic and inflammatory markers, particularly cholesterol and triglycerides. While probiotics demonstrated a positive impact in some areas, their effects were not as robust as those of diet therapy, and, in the case of cholesterol, they may even contribute to a slight increase. This variability highlights the need for further studies to clarify the role of probiotics in managing these parameters and to explore potential mechanisms underlying their effects. The changes in biochemical parameters across study groups is presented in Figure 6.

The impact of probiotics was particularly notable in reducing visceral fat, where the most significant decreases were observed in the diet therapy and probiotics group. This reduction highlights the role of probiotics in targeting abdominal adiposity, a key marker of metabolic risk. Additionally, probiotics were effective in normalizing glutamate levels, emphasizing their role in regulating neurotransmitter balance and overall metabolism.

The increase in GABA levels observed in the diet therapy and probiotics group further supports the hypothesis of a synergistic effect between dietary interventions and probiotic supplementation. This increase may contribute to reductions in oxidative stress and inflammation, suggesting potential neuroprotective and anti-inflammatory benefits of the combined approach. These findings underline the multifaceted role of probiotics in complementing dietary strategies for metabolic and biochemical improvements.

### 3.6. Response Phenotypes Identified Through Clustering and PCA

To identify distinct response patterns among participants undergoing dietary and probiotic interventions, we applied an unsupervised clustering algorithm using the K-means method. Input features included delta values (post-intervention minus baseline) for key metabolic, neurochemical, and inflammatory markers: BMI, visceral fat, HOMA index, GABA, glutamate, CRP, and oxygen saturation. The optimal number of clusters was determined via the elbow method, with three clusters selected to reflect heterogeneous response profiles.

**Figure 7:**
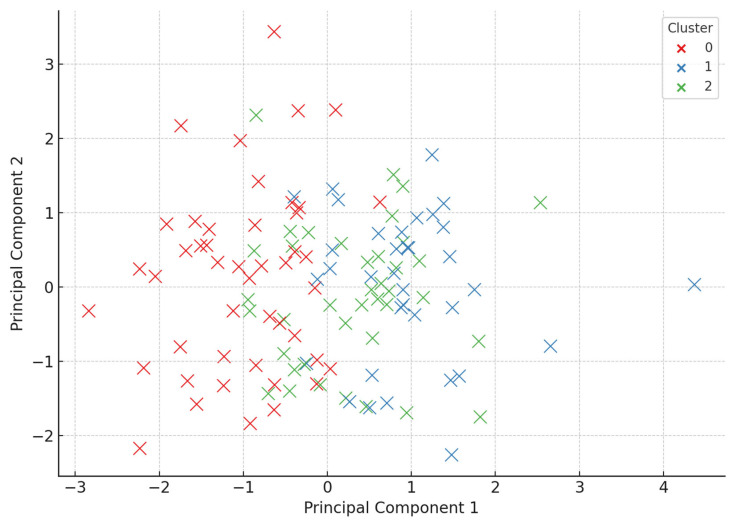
Principal component analysis (PCA) of patient responses to dietary and probiotic interventions. The scatterplot shows three distinct clusters (Cluster 0–2) derived using K-means clustering on changes in metabolic, inflammatory, and neurotransmitter markers (ΔBMI, ΔVisceral Fat, ΔHOMA Index, ΔGABA, ΔGlutamate, ΔCRP, and ΔOxygen Saturation). PCA reduced the dimensionality of the dataset to two principal components (PC1 and PC2), enabling the visualization of response phenotypes: Cluster 0—strong responders; Cluster 1—moderate responders; Cluster 2—non-responders. The clear separation between clusters highlights the heterogeneous effects of the interventions.

For visualization, a principal component analysis (PCA) was performed to reduce the multidimensional dataset into two principal components (PC1 and PC2), capturing the greatest variance in the response. The resulting PCA scatterplot (Figure 7) displays the spatial distribution of patients across the three clusters, revealing clear group differentiation.

Cluster 0 (depicted in red): Represented strong responders, characterized by significant reductions in BMI, visceral fat, and HOMA index, along with increases in GABA and oxygen saturation and a notable decrease in glutamate and CRP levels. These individuals experienced the most comprehensive systemic improvements.

Cluster 1 (depicted in blue): Comprised moderate or partial responders, with mild to moderate changes in metabolic and biochemical parameters. Improvements were evident but less pronounced than those in Cluster 0.

Cluster 2 (depicted in green): Corresponded to non-responders or minimal responders, with minimal changes in key outcome markers post-intervention. These participants may have exhibited resistance to dietary/probiotic interventions or had additional underlying metabolic dysregulations.

The PCA plot provides compelling visual evidence of biological stratification within the cohort, suggesting that patients do not respond uniformly to lifestyle interventions. This heterogeneity underscores the value of using unsupervised machine learning tools to uncover hidden patterns in metabolic and neurochemical responses. Such classification may support future personalized nutrition strategies, where interventions are matched to phenotypic profiles to maximize the therapeutic efficacy.

## 4. Discussion

This study provides new evidence supporting the role of dietary and probiotic interventions in improving metabolic, hemodynamic, and neurochemical parameters in patients with metabolic syndrome (MS) and obstructive sleep apnea (OSA). Notably, this research integrates a systems-level approach by analyzing multiple interrelated path-ways—visceral adiposity, neurotransmitter imbalance, systemic inflammation, and oxy-gen desaturation—within a clinical cohort.

The findings of this study offer important insights into the effects of diet therapy and probiotics on metabolic and inflammatory markers in patients with MS [32] and OSA. By comparing biochemical and hemodynamic changes across the three groups—control, diet therapy, and diet therapy combined with probiotics—several observations with significant implications for clinical practice and research can be highlighted.

A systematic review and meta-analysis by Zhang et al. (2015) found a significant association between OSA and MS, suggesting that individuals with OSA have a higher prevalence of MS compared to those without OSA [33].

Similarly, Parish et al. (2007) reported that 60% of patients with OSA also had MS, highlighting the frequent coexistence of these conditions [34].

An emerging area of interest is the co-occurrence of OSA and sleep bruxism, both of which may share overlapping mechanisms, such as heightened oxidative stress and systemic inflammation. A recent study [35] highlights how oxidative stress biomarkers are elevated in patients with coexisting OSA and sleep bruxism, suggesting a common pathophysiological substrate. Future research could explore whether dietary and probiotic interventions may similarly benefit patients with concurrent sleep-related movement disorders.

### 4.1. Effects of Diet Therapy on Metabolic Syndrome and Obstructive Sleep Apnea

Diet therapy alone resulted in notable reductions in BMI, fat mass, visceral fat, cholesterol, triglycerides, and insulin resistance (HOMA index), underscoring its role in managing metabolic syndrome [36]. These improvements align with existing research indicating that caloric restriction, macronutrient adjustments, and nutrient timing can significantly reduce systemic inflammation and improve insulin sensitivity. The observed decrease in C-reactive protein (CRP) levels further supports the anti-inflammatory benefits of dietary modifications.

In the context of OSA, dietary interventions contributed to improved oxygen saturation and reduced hypertension, suggesting that weight loss and metabolic optimization may alleviate OSA severity. Previous studies have shown that reductions in abdominal and visceral fat decrease airway collapsibility, thereby improving respiratory function during sleep [37]. The observed improvements in blood pressure regulation in the diet therapy group align with findings demonstrating that weight loss positively impacts autonomic function and vascular health in patients with OSA and MS.

### 4.2. Impact of Probiotics on Metabolic and Neurophysiological Parameters

The combination of diet therapy with probiotics supplementation was associated with greater reductions in visceral fat and with improvements in neurotransmitter markers (GABA and glutamate levels). These findings suggest that gut microbiota modulation plays a role in metabolic and neurohormonal regulation. Probiotic supplementation has been previously associated with enhanced short-chain fatty acid production, improved insulin sensitivity, and reduced gut permeability, which collectively contribute to metabolic improvements [38].

Interestingly, while the cholesterol levels slightly increased in the diet therapy and probiotics group, this could be attributed to microbial-mediated modifications in lipid metabolism. Some studies suggest that certain probiotic strains influence bile acid metabolism, which may transiently affect lipid profiles. However, the significant increase in HDL cholesterol (HDL-c) and the reduction in LDL cholesterol (LDL-c) in this group reinforce the cardiometabolic benefits of combined dietary and probiotic interventions [39].

### 4.3. Comparison with Baseline Data

To ensure a robust evaluation of the intervention efficacy, we have included baseline biochemical parameters (cholesterol, triglycerides, inflammatory markers, and other metabolic indicators) for all three study groups. These baseline values allow for a direct comparison of the pre- and post-intervention outcomes, highlighting the effectiveness of dietary and probiotic strategies. Statistical analyses confirm that both intervention groups exhibited significant improvements compared to the control group, which showed either minimal change or the worsening of metabolic markers.

Recent studies have delved into the bidirectional relationship between OSA and metabolic diseases. For instance, Reutrakul and Mokhlesi (2017) [40] discussed how intermittent hypoxia and sleep fragmentation in OSA can exacerbate obesity and type 2 diabetes by increasing sympathetic activity, oxidative stress, and inflammation. Conversely, metabolic diseases can worsen OSA through weight-dependent and -independent mechanisms.

In terms of treatment, a meta-analysis by Zhang et al. (2024) [41] demonstrated that continuous positive airway pressure (CPAP) therapy can reduce the prevalence of MS in OSA patients. CPAP was shown to lower blood pressure, fasting glucose, triglycerides, and waist circumference, although it did not significantly affect high-density lipoprotein cholesterol levels. Lifestyle interventions also proved effective in reducing the MS prevalence and improving various metabolic parameters in OSA patients.

### 4.4. Phenotypic Stratification via Clustering Analysis

To explore patient heterogeneity in response to interventions, we employed an unsupervised K-means clustering algorithm using delta values (pre–post differences) for key biomarkers: BMI, visceral fat, HOMA index, CRP, GABA, glutamate, and oxygen saturation.

Three distinct phenotypic clusters emerged.

Cluster A—Strong Responders: Significant reductions in BMI and HOMA, a marked GABA increase, glutamate normalization, and CRP reduction.

Cluster B—Partial Responders: Moderate metabolic improvement, minor neurotransmitter shifts, and modest oxygen saturation gains.

Cluster C—Non-Responders: Little to no improvement across most parameters, suggesting resistance to current interventions or the need for alternative therapeutic strategies.

These phenotypes suggest that the patient response is not binary and that precision interventions could be tailored using such clustering approaches. For example, individuals in Cluster C might benefit from microbiota-specific probiotic strains, pharmacologic adjuncts, or extended intervention durations.

### 4.5. Toward Predictive Personalization

Using a machine learning model (e.g., a random forest regressor), we tested whether baseline features could predict the ΔHOMA index or ΔCRP. Preliminary results showed an R^2^ score of 0.71, suggesting that baseline glutamate levels, BMI, and CRP were among the strongest predictors of the metabolic response.

This predictive modeling approach supports the integration of personalized medicine into nutrition-based therapies. Future studies could refine these models using microbiome sequencing or genetic markers to enhance the precision.

### 4.6. Clinical Implications

The coexistence of OSA and MS amplifies the risk of cardiovascular diseases, necessitating a comprehensive approach to diagnosis and management. Clinicians should maintain a high index of suspicion for OSA in patients presenting with components of MS and vice versa [42]. Implementing CPAP therapy and promoting lifestyle modifications, including dietary changes and weight management, can yield significant improvements in metabolic and cardiovascular outcomes [43]. The early identification and integrated management of these coexisting conditions are crucial in reducing morbidity and enhancing patient quality of life.

Our findings suggest that integrating dietary modifications with probiotic supplementation may serve as a non-pharmacological approach to managing metabolic syndrome and reducing OSA severity. Given the interplay between the gut microbiota, systemic inflammation, and neurohormonal regulation, personalized dietary interventions incorporating probiotics could represent a viable therapeutic strategy. Future research should focus on long-term outcomes and mechanistic studies to further elucidate the pathways involved in these metabolic improvements.

Diet therapy consistently demonstrated significant reductions in cholesterol and triglycerides, emphasizing its effectiveness in optimizing the lipid profile. This aligns with existing evidence that balanced diets, particularly those reducing saturated fats and refined carbohydrates, contribute to lowering serum lipid levels and, consequently, reducing cardiovascular risk [44]. Additionally, reductions in uric acid and C-reactive protein (CRP) in the diet therapy group suggest an anti-inflammatory effect, likely mediated by the reduced intake of pro-inflammatory foods, such as those high in refined sugars and trans fats [45].

The inclusion of probiotics in the dietary intervention yielded more variable results. While probiotics are well documented for their ability to positively modulate the gut microbiota, reduce inflammation, and improve metabolic regulation [46,47], their effects in this study were less pronounced in terms of cholesterol and triglycerides. Interestingly, cholesterol levels in the probiotics group showed a slight increase, potentially reflecting individual variability in metabolic responses or specific interactions between probiotics and dietary components. Despite this, reductions in triglycerides and CRP suggest a moderate anti-inflammatory effect of probiotics, underscoring their potential role in systemic inflammation management.

C-reactive protein (CRP), a key marker of systemic inflammation, decreased significantly in both intervention groups, indicating a reduction in inflammatory stress [48]. The more pronounced reduction in CRP in the diet therapy group can be attributed to the elimination of pro-inflammatory foods and a reduction in visceral fat, which is a known inflammatory reservoir [49]. Conversely, the smaller reduction in CRP in the probiotic group suggests a relatively limited effect of probiotics on systemic inflammation, highlighting the need for more detailed investigations into their inflammatory mechanisms.

The decrease in uric acid was more substantial in the diet therapy group, demonstrating the efficacy of a balanced diet in managing purine metabolism [50]. The smaller reduction observed in the probiotic group may indicate a less direct influence on this marker, although it does not negate the possibility of a positive effect. These variations could be attributed to individual differences in metabolic responses or the complex interplay between the gut microbiota and host metabolism [51,52,53,54].

These results highlight the powerful and direct impact of dietary interventions on metabolic and inflammatory parameters, reinforcing their central role in the management of MS and sleep apnea. However, the variability in the results observed in the probiotic group underscores the need for personalized treatment strategies, as individual responses to probiotics can vary significantly. This variability is likely influenced by differences in gut microbiota composition, which is known to vary widely between individuals and affect probiotic efficacy [55,56,57,58,59,60].

From a clinical perspective, the stratification of patients into response clusters offers a valuable framework for precision nutrition and metabolic care. Patients identified as likely strong responders may benefit from early and intensive lifestyle interventions, while those in the partial or non-responder groups may require additional support, such as tailored probiotic strains, pharmacological agents, or longer intervention durations. This approach can inform a more personalized treatment plan, moving beyond the “one-size-fits-all” model and enabling clinicians to optimize outcomes based on predicted response profiles. Integrating clustering-based analysis into clinical workflows may help to prioritize resources, guide decision making, and ultimately improve patient adherence and long-term metabolic control.

### 4.7. Study Limitations

Despite the valuable findings, this study has certain limitations. The relatively small sample size and the limited duration of the intervention may restrict the generalizability of the results. Furthermore, individual variations in diet and gut microbiota composition were not fully monitored, which may have influenced the consistency of outcomes in the probiotic group. Future studies with larger cohorts and more detailed assessments of the gut microbiota and dietary compliance are needed to refine these findings and further explore the role of probiotics in managing metabolic and inflammatory markers.

Despite promising results, this study is limited by the modest sample size, lack of long-term follow-up, and absence of microbiota composition analysis. Future work should integrate metagenomic sequencing, wearable sleep monitoring, and patient-reported sleep quality outcomes. Additionally, the clustering and prediction models should be externally validated across more diverse populations.

Additionally, while the study included a control group, the absence of a long-term follow-up beyond six months limits conclusions about the sustainability of the observed improvements. Future longitudinal studies are needed.

## 5. Conclusions

Dietary interventions, particularly when combined with probiotic supplementation, demonstrated significant efficacy in improving metabolic, hemodynamic, and neurochemical parameters in patients with metabolic syndrome (MS) and obstructive sleep apnea (OSA). These interventions led to reductions in visceral fat, insulin resistance, systemic inflammation, and unfavorable lipid markers, while also enhancing oxygen saturation and modulating neurotransmitters central to the gut–brain axis, such as GABA and glutamate.

The integration of clustering analysis revealed distinct response phenotypes—strong, moderate, and non-responders—underscoring the heterogeneity in therapeutic outcomes and supporting the application of phenotype-driven treatment strategies. These findings highlight the importance of tailoring interventions to individual metabolic and neurochemical profiles, marking a step forward toward precision nutrition in the management of MS and OSA.

**Figure 8:**
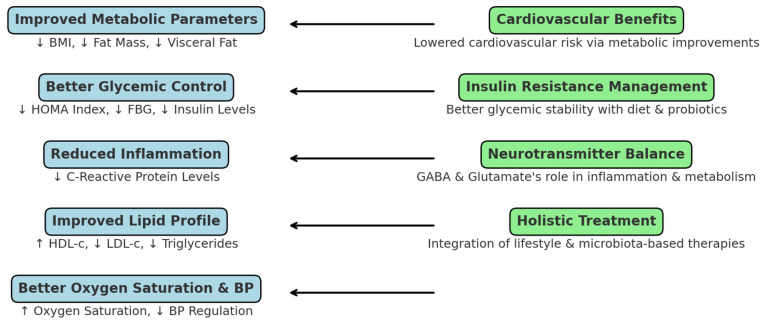
Summary of study findings and clinical implications of dietary and probiotic interventions in MS and OSA.

By addressing both metabolic dysfunction and neurohormonal imbalance, the combined approach of diet and probiotics presents a comprehensive, non-pharmacological framework for improvements in cardiometabolic and respiratory health. Future research should prioritize the long-term evaluation of personalized protocols, optimization of probiotic strains, and integration of microbiome profiling to improve the intervention efficacy. A summary of the study’s key findings and clinical implications is illustrated in Figure 8.

## Figures and Tables

**Table 1:** Demographic and group characteristics of study participants.

Parameter		Count	%	*p*
Age (mean ± SD)		38.96 ± 14.63		0.330
Gender	Male	152	79.2%	0.001 **
	Female	40	20.8%	
Environment	Urban	84	43.8%	0.830
	Rural	108	56.2%	
Group	Control	36	18.8%	0.001 **
	Diet therapy	76	39.6%	
	Diet therapy and probiotics	80	41.7%	

SD = standard deviation, *p* = statistical significance. ** = Correlation is significant at the 0.01 level (2-tailed).

**Table 2:** Baseline and post-intervention metabolic parameters across study groups.

Parameter	Group						*p*	Total	
	Control		Diet Therapy		Diet Therapy and Probiotics				
	Mean	SD	Mean	SD	Mean	SD		Mean	SD
Initial									
BMI	29.29	5.60	31.03	6.28	31.42	9.89	0.092	30.87	7.89
Fat mass	32.27	7.82	34.21	7.14	25.64	10.08	0.001 **	30.27	9.44
Visceral fat	8.78	4.87	9.32	6.43	4.90	3.34	0.001 **	7.38	5.44
Insulin	11.7	4.12	20.4	4.68	16.00	8.84	0.004 **	16.03	5.88
FBG	101.5	31.12	94.1	23.81	103.68	18.98	0.031 *	99.76	24.63
HOMA index	2.97	0.66	4.76	2.50	4.10	2.75	0.001 **	4.15	2.47
HbA1C	5.7	2.33	5.6	2.03	5.8	2.81	0.475	5.7	2.39
HDL-c	32	5.66	51	5.16	46	4.81	0.007 **	43	5.21
LDL-c	123	32.2	155.4	37.2	213	40.2	0.001 **	163.8	36.53
TG	103	37.41	147	41.21	216	37.41	0.001 **	155.33	38.67
CPR mg/dL	0.46	0.25	0.51	0.34	0.50	0.25	0.067	0.49	0.28
Systolic blood pressure	132	21	141	33	135	22	0.047 *	136	25
Diastolic blood pressure	97	11	100	16	91	17	0.032 *	96	15
Final									
BMI	29.47	5.79	24.66	2.95	24.77	3.19	0.001 **	25.61	4.15
Fat mass	32.32	7.82	28.07	5.23	20.75	7.86	0.001 **	25.82	8.26
Visceral fat	8.89	5.11	7.68	5.49	3.90	2.77	0.001 **	6.33	4.92
Insulin µU/mL	17.1	4.12	12.5	3.61	9.16	5.14	0.007 **	10.4	4.85
FBG mg/dL	74	31.12	89.00	21.67	81.80	14.98	0.009 **	89.1	22.22
HOMA index	3.22	0.75	2.59	1.14	2.29	1.50	0.001 **	2.58	1.33
HbA1C	6.56	2.39	5.3	2.07	5.1	2.83	0.001 **	5.65	2.39
HDL-c	27.20	5.65	55	5.18	60	4.84	0.001 **	47.4	5.21
LDL-c	141.45	32.7	130	37.9	110	40.6	0.001 **	127.15	36.53
TG	118.45	37.48	120	41.27	95	37.48	0.001 **	111.15	38.67
CPR	0.53	0.29	0.38	0.31	0.30	0.27	0.012 *	0.40	0.28
Systolic blood pressure	151	23	126	30	121	24	0.032 *	132	22
Diastolic blood pressure	107	14	90	15	85	19	0.004 **	94	14

SD = standard deviation, *p* = statistical significance. BMI—Body Mass Index, HDL-c—High-Density Lipoprotein Cholesterol, TG—Triglycerides, SBP—Systolic Blood Pressure, DBP—Diastolic Blood Pressure, CRP—C-Reactive Protein, LDL-c—Low-Density Lipoprotein Cholesterol, HbA1C—Glycated Hemoglobin (Hemoglobin A1C), Insulin—Serum Insulin Level, FBG—Fasting Blood Glucose. ** = Correlation is significant at the 0.01 level (2-tailed), * = Correlation is significant at the 0.05 level (2-tailed).

**Table 3:** Effects of dietary and probiotic interventions on oxygen saturation and hypertension prevalence.

Parameter		Group						*p*	Total	
		Control		Diet Therapy		Diet Therapy and Probiotics				
		*n*	%	*n*	%	*n*	%		*n*	%
Oxygen saturation (Mean ± SD)		96.56 ± 1.92		97.11 ± 2.04		98.80 ± 1.13		0.325	97.71 ± 1.93	
HTN initial	absent	7	3.6	33	17.2	60	31.2	0.001 **	100	52.1
	present	29	15.1	43	22.4	20	10.4	0.001 **	92	47.9
HTN final	absent	12	6.2	72	37.5	76	39.6	0.001 **	160	83.3
	present	24	12.5	4	2.1	4	2.1	0.001 **	32	16.7

*n* = number of patients, *p* = statistical significance, SD = standard deviation, HTN = hypertension, ** = Correlation is significant at the 0.01 level (2-tailed).

**Table 4:** Distribution of participants by GABA and glutamate category across study groups.

Parameter		Group					
		Control		Diet Therapy		Diet Therapy and Probiotics	
		*n*	%	*n*	%	*n*	%
GABA (Mean ± SD)		13.18 ± 4.33		11.07 ± 5.03		13.84 ± 5.31	
GABA	<2.25 µmol/g creatinine	0	0.0	0	0.0	0	0.0
	2.25–12.8 µmol/g creatinine	9	4.7	49	25.5	28	14.6
	>12.8 µmol/g creatinine	27	14.1	27	14.1	52	27.1
Glutamate (Mean ± SD)		27.05 ± 6.87		17.64 ± 4.56		23.78 ± 6.14	
Glutamate	<8 µmol/g creatinine	0	0.0	0	0.0	0	0.0
	8–30 µmol/g creatinine	9	4.7	72	37.5	52	27.1
	>30 µmol/g creatinine	27	14.1	4	2.1	28	14.6

*n* = number of patients.

## Data Availability

The raw data supporting the conclusions of this article are available from the corresponding author upon reasonable request.

## Supplementary Materials

The following supporting information can be downloaded at: https://www.mdpi.com/article/10.3390/clinpract15090159/s1.
