# Supplementary material for: Effects of Dietary and Probiotic Interventions in Patients with Metabolic Syndrome and Obstructive Sleep Apnea
Source: Clin Pract. 2025 Aug 29;15(9):159. doi: 10.3390/clinpract15090159 (PMC12468276; doi:10.3390/clinpract15090159)
Supplement: Supplementary file 1 [file clinpract-15-00159-s001.zip › clinpract-3678351-supplementary.pdf]

Group	Age (years)	Sex (M/F)	BMI (kg/m²)	Hypertension (%)	Diabetes (%)	Dyslipidemia (%)	OSA severity (AHI)	CPAP use (%)	On statins (%)	On antidiabetics (%)	On antihypertensives (%)	Duration of MS (years)	Duration of OSA (years)	Prior diet therapy (%)	Prior probiotic use (%)
Control	60	M	28	1	1	0	28.9	0	0	1	1	1.4	5.1	0	0
Control	53.6	M	25.7	1	0	1	21.9	0	0	1	0	2.6	5.7	0	0
Control	61.5	F	35.1	0	1	1	33.2	1	0	0	1	3.7	6.3	0	0
Control	70.2	M	29.8	1	1	1	34.6	1	0	1	1	7.3	3	0	0
Control	52.7	F	26.6	1	0	0	31.7	1	0	1	1	8.1	2.7	0	0
Control	52.7	F	28.5	0	1	1	23.2	1	1	1	0	4.1	6.7	0	0
Control	70.8	F	27.5	0	0	1	24.2	0	0	1	1	4.2	-0.1	0	0
Control	62.7	F	32.5	0	0	1	22.3	1	0	0	0	5.5	7.1	0	0
Control	50.3	F	34.3	0	0	0	31.8	1	0	0	1	5	3.4	0	0
Control	60.4	F	35.6	1	1	1	34.3	0	1	0	1	6.9	4	0	0
Control	50.4	F	35.6	1	1	0	30.1	1	0	1	0	4	2.6	0	0
Control	50.3	F	35	1	0	1	32.7	1	1	0	1	6.9	3.9	0	0
Control	57.4	F	31.2	1	0	1	23.7	1	0	0	1	4.5	6.5	0	0
Control	35.9	F	29.5	1	1	1	28.7	1	0	0	0	6.7	5.5	0	0
Control	37.8	F	26	0	0	1	21.2	1	0	0	0	4.5	5	0	0
Control	49.4	M	36.4	1	0	1	24.1	0	0	1	0	3.8	4.2	0	0
Control	44.9	M	31.3	1	1	1	32.4	0	1	1	0	8.8	1.1	0	0
Control	58.1	F	30.4	1	1	1	26.8	1	1	0	0	7.1	2.3	0	0
Control	45.9	F	36.7	1	0	1	34	1	1	0	1	1.4	3.3	0	0
Control	40.9	F	28.4	1	1	0	30.7	1	0	0	0	4.1	4.9	0	0
Control	69.7	F	35.3	0	1	1	41.7	1	0	0	0	7.4	4.1	0	0
Control	52.7	F	33.2	0	0	1	28.5	1	1	0	1	2.6	6	0	0
Control	55.7	F	27.8	1	1	1	21	1	0	0	0	6.5	4.9	0	0
Control	40.8	F	31.7	1	0	1	27.8	1	0	0	1	6.3	4.1	0	0
Control	49.6	F	35.9	1	1	1	23.2	1	0	1	0	3.6	8.6	0	0
Control	56.1	M	35.2	0	1	1	32.9	1	0	1	0	5	4.8	0	0
Control	43.5	F	38	1	1	1	28.2	1	1	0	1	0.8	3.1	0	0
Control	58.8	M	33.4	1	0	1	27.3	1	1	0	1	8.5	7.7	0	0

Control	49	F	45.5	0	1	1	27.4	0	1	1	1	4.4	6.4	0	0
Control	52.1	F	28.3	0	0	1	31.7	0	0	1	1	6.6	7.6	0	0
Control	49	M	30.4	1	0	0	25.7	0	0	1	1	6.2	3.8	0	0
Control	73.5	F	31.8	1	0	0	31.9	1	0	1	0	7.7	6	0	0
Control	54.9	M	26.3	1	1	1	33.2	0	0	0	0	4.1	1.1	0	0
Control	44.4	F	36.2	1	1	1	26.3	0	0	0	1	5.4	5.6	0	0
Diet	53.4	F	30.1	1	0	1	26.8	1	0	0	0	4.7	6.1	0	0
Diet	41.4	M	37.3	1	0	1	32.1	0	0	0	1	6.6	4.2	0	0
Diet	65.3	F	35.9	1	0	1	32.5	1	0	0	0	5.8	5.6	0	0
Diet	36.9	F	33.3	0	0	1	28.5	1	1	1	0	0.3	5.2	0	0
Diet	35.9	M	31.9	0	0	1	23.6	1	0	1	1	4.3	6.6	0	0
Diet	58.6	F	35.2	1	0	1	29.4	1	0	0	1	5.4	6.1	0	0
Diet	60.7	F	30.9	1	1	1	28.8	1	1	0	0	2.1	3.1	0	0
Diet	64.7	M	25.3	1	0	1	30.8	1	0	0	1	6.2	5.3	0	0
Diet	62.4	M	32	1	1	1	23.6	0	1	1	0	2	0.9	0	0
Diet	69.8	M	33.6	0	1	1	26	1	0	0	1	3.4	3.9	0	0
Diet	62	F	36.5	1	1	1	30.5	1	1	0	1	7.7	5.6	0	0
Diet	53.7	M	26.2	1	0	1	34.2	0	0	1	0	3.2	4.3	0	0
Diet	54.3	M	31.5	0	0	1	32.8	1	0	0	0	6.1	4.9	0	0
Diet	52.2	M	31.7	0	1	0	28.4	1	0	1	0	1.5	3.6	0	0
Diet	58	M	27.6	1	1	1	25.6	1	1	0	1	9.2	6.3	0	0
Diet	44	M	34	0	1	1	30	0	0	0	1	4.4	3.8	0	0
Diet	58.4	M	35	1	0	0	22.5	0	0	0	0	5.2	3.1	0	0
Diet	54.1	F	30.8	0	0	0	23.9	1	0	1	0	5.4	5.8	0	0
Diet	57.8	F	40.2	1	1	1	35.5	1	1	1	1	6.2	1.2	0	0
Diet	57.8	M	30.7	0	1	1	30	0	0	1	1	2	5	0	0
Diet	30.5	M	30.2	1	1	1	26.3	1	1	0	1	5	1.9	0	0
Diet	46	M	30.1	1	1	0	32.1	1	0	1	0	3.7	6.6	0	0
Diet	53.9	F	28.7	1	0	1	34.4	1	0	0	0	1.6	3.5	0	0
Diet	54.4	F	29.8	1	1	1	25.8	1	0	0	0	7	4.3	0	0

Diet	57.6	F	26.3	1	0	0	25.9	0	0	1	1	4.3	4.8	0	0
Diet	58	F	31.2	0	0	0	25.2	0	0	0	0	5.5	0.1	0	0
Diet	70.2	F	32.2	1	1	1	32.8	0	0	0	1	5.4	2.7	0	0
Diet	58.1	F	22.9	1	1	1	32.9	1	1	0	0	4.3	6.1	0	0
Diet	51.9	F	31.6	1	0	1	34.9	0	0	0	1	9	5.7	0	0
Diet	39	F	32.2	1	0	1	33	0	0	0	1	4.9	4.6	0	0
Diet	41.3	F	34.1	0	1	1	19.8	0	0	1	1	1.9	1.3	0	0
Diet	60.4	M	36.8	0	0	1	29.4	1	0	0	0	4.2	5.2	0	0
Diet	65.6	M	35.4	1	1	1	22.7	1	0	1	0	6	5.4	0	0
Diet	70.4	M	29.5	1	0	1	25.4	0	0	1	0	6.1	4.1	0	0
Diet	46.7	M	29.1	1	1	1	28.9	0	0	0	1	2.8	6.4	0	0
Diet	52	F	34.3	1	1	1	32.2	1	1	0	1	6.9	2.6	0	0
Diet	57.4	F	29.8	1	0	1	23.5	0	1	0	1	1.5	3	0	0
Diet	75.5	M	27.1	1	0	1	16.4	0	0	1	1	7.3	-1	0	0
Diet	56.1	F	41.5	0	0	1	28.9	0	0	0	1	2.4	5.8	0	0
Diet	70.5	M	30.8	0	0	1	41.2	0	1	1	0	4.5	4.6	0	0
Diet+Probiotic	38.2	F	36.8	1	1	1	37.1	0	0	0	0	4.6	5.6	0	0
Diet+Probiotic	37.4	M	33.4	0	1	0	29.8	1	0	1	1	6.1	2.9	0	0
Diet+Probiotic	46.3	M	37.3	1	1	0	30	1	1	0	0	4.9	7.6	0	0
Diet+Probiotic	62.9	M	27.5	1	0	1	31.4	1	1	1	0	3.1	2.3	0	0
Diet+Probiotic	52	M	25.4	0	0	1	25.1	0	0	0	0	3.3	4.5	0	0
Diet+Probiotic	47	F	33.9	1	1	1	33.4	1	1	1	0	3	2.4	0	0
Diet+Probiotic	44.9	F	33	1	1	1	28.6	1	1	1	1	6.9	8.4	0	0
Diet+Probiotic	44.5	F	33.2	0	1	1	32.8	0	0	1	0	3.8	1.5	0	0
Diet+Probiotic	35.7	M	35.7	1	0	1	27	0	1	1	0	1.2	5	0	0
Diet+Probiotic	42.9	M	33.7	1	0	1	34.6	1	0	0	0	5.8	2.5	0	0

Diet+Probiotic	44.3	F	33.6	1	0	0	30	0	0	1	0	8	3.3	0	0
Diet+Probiotic	61.8	M	34.9	0	1	1	38.9	1	0	1	0	6.5	4.9	0	0
Diet+Probiotic	53.3	M	34	1	0	0	26.2	0	0	1	0	3.8	6.4	0	0
Diet+Probiotic	46.7	M	35.4	1	1	1	29	0	0	0	1	1.8	7.2	0	0
Diet+Probiotic	78.1	M	36	1	1	1	22.8	1	1	1	1	4.7	0.9	0	0
Diet+Probiotic	48.4	F	38.1	1	1	0	25.4	0	0	1	1	4.7	4.5	0	0
Diet+Probiotic	66.7	F	29.4	0	0	1	31.1	1	0	1	0	3.1	6.9	0	0
Diet+Probiotic	71.2	F	33.9	1	1	1	33.6	0	0	0	0	6.1	0.6	0	0
Diet+Probiotic	39.3	M	30.5	0	1	1	42.8	1	1	1	1	8.7	4.6	0	0
Diet+Probiotic	55.7	F	28.7	0	1	1	27.5	0	0	0	1	5.4	4	0	0
Diet+Probiotic	49.8	F	29.2	1	1	0	23.3	0	0	1	0	5.3	4.8	0	0
Diet+Probiotic	49	M	34	1	1	1	31.8	1	0	0	0	3.1	2.5	0	0
Diet+Probiotic	60.6	M	33.7	1	1	1	22.4	0	0	1	0	7.8	1.1	0	0
Diet+Probiotic	48.8	F	33.6	1	0	0	30.8	1	1	1	0	4.8	0.2	0	0
Diet+Probiotic	50	F	31.1	1	0	1	25.5	0	1	0	0	3.8	6	0	0
Diet+Probiotic	53.3	F	32	0	1	0	29.1	1	0	1	1	5.1	6.2	0	0
Diet+Probiotic	51.3	F	35.3	1	1	0	33.5	0	1	0	1	5.9	4.6	0	0
Diet+Probiotic	43.3	M	26	1	1	1	27.4	1	1	1	1	5.3	2.9	0	0
Diet+Probiotic	63	F	32.1	1	1	0	32.7	0	1	1	1	7.4	0.8	0	0
Diet+Probiotic	65.5	F	32.4	0	0	1	32.4	0	0	1	1	5.8	5.1	0	0
Diet+Probiotic	58.4	M	35	1	0	1	18.7	0	0	1	1	4.4	1.8	0	0

Diet+Probiotic	51.2	F	26.6	1	1	1	23.5	0	0	1	1	5.8	2.6	0	0
Diet+Probiotic	47.9	M	38	0	1	1	20.2	1	1	0	0	6	2.8	0	0
Diet+Probiotic	48	M	32.9	0	0	0	38	0	0	0	0	2.4	3.6	0	0
Diet+Probiotic	78.6	M	33.1	1	1	0	27.5	1	0	1	0	7.3	2.5	0	0
Diet+Probiotic	64.4	F	30.4	1	0	1	36.9	0	0	1	1	4.4	4.1	0	0
Diet+Probiotic	46.6	F	34.2	0	1	1	19.8	0	1	1	1	4.4	2.8	0	0
Diet+Probiotic	56.6	M	32.2	1	0	0	25.1	1	0	1	0	6.5	5.4	0	0
Diet+Probiotic	51.4	F	36.8	0	1	1	32	1	1	0	1	6.5	6.1	0	0
Diet+Probiotic	60.8	F	26.2	1	0	0	28.9	0	1	1	0	3.6	4.2	0	0
